# “Enhanced recovery after surgery – ERAS in elective craniotomies-a non-randomized controlled trial”

**DOI:** 10.1186/s12883-021-02150-7

**Published:** 2021-03-19

**Authors:** Anirudh Elayat, Sritam S. Jena, Sukdev Nayak, R. N. Sahu, Swagata Tripathy

**Affiliations:** 1 All India Institute of Medical Sciences, Bhubaneswar, India; 2 Department of Anesthesia and Critical Care, AIIMS Bhubaneswar, Bhubaneswar, India; 3 Department of Neurosurgery, AIIMS Bhubaneswar, Bhubaneswar, India; 4 Fellow Neuroanesthesia, Walton Centre, Liverpool, UK

**Keywords:** Enhanced recovery after surgery (ERAS), Complex carbohydrate, Glycemic control, Neurosurgery and neurocritical care, Perioperative care bundle, Pre-emptive analgesia

## Abstract

**Background:**

Enhanced Recovery After Surgery (ERAS) is a multimodal perioperative care bundle aimed at the early recovery of patients. Well accepted in gastric and pelvic surgeries, there is minimal evidence in neurosurgery and neurocritical care barring spinal surgeries. We wished to compare the length of intensive care unit (ICU) or high dependency unit (HDU) stay of patients undergoing elective craniotomy for supratentorial neurosurgery: ERAS protocol versus routine care. The secondary objective was to compare the postoperative pain scores, opioid use, glycemic control, and the duration of postoperative hospital stay between the two groups.

**Methods:**

In this pragmatic non-randomized controlled trial (CTRI/2017/07/015451), consenting adult patients scheduled for elective supratentorial intracranial tumor excision were enrolled prospectively after institutional ethical clearance and consent. Elements-of-care in the ERAS group were- Preoperative –family education, complex-carbohydrate drink, flupiritine; Intraoperative – scalp blocks, limited opioids, rigorous fluid and temperature regulation; Postoperative- flupiritine, early mobilization, removal of catheters, and initiation of feeds. Apart from these, all perioperative protocols and management strategies were similar between groups. The two groups were compared with regards to the length of ICU stay, pain scores in ICU, opioid requirement, glycemic control, and hospital stay duration. The decision for discharge from ICU and hospital, data collection, and analysis was by independent assessors blind to the patient group.

**Results:**

Seventy patients were enrolled. Baseline demographics – age, sex, tumor volume, and comorbidities were comparable between the groups. The proportion of patients staying in the ICU for less than 48 h after surgery, the cumulative insulin requirement, and the episodes of VAS scores > 4 in the first 48 h after surgery was significantly less in the ERAS group – 40.6% vs. 65.7%, 0.6 (±2.5) units vs. 3.6 (±8.1) units, and one vs. ten episodes (*p* = 0.04, 0.001, 0.004 respectively). The total hospital stay was similar in both groups.

**Conclusion:**

The study demonstrated a significant reduction in the proportion of patients requiring ICU/ HDU stay > 48 h. Better pain and glycemic control in the postoperative period may have contributed to a decreased stay. More extensive randomized studies may be designed to confirm these results.

**Trial registration:**

Clinical Trial Registry of India (CTRI/2018/04/013247), registered retrospectively on April 2018.

## Background

Enhanced Recovery After Surgery (ERAS) is a multimodal perioperative care pathway that has led to a dramatic change in the conventional surgical doctrine [1]. Its implementation has demonstrated success in early rehabilitation, and shortened hospital stay and Intensive Care Unit (ICU) stay for postoperative patients undergoing elective gastroenterological, urological, obstetric, and spine and liver procedures [2–6].

ERAS protocols target perioperative stress response with specific goal-directed evidence-based practices [7]. Many surgical-disciplines have developed modifications of ERAS protocols to improve patient compliance and achieve better results. Decreased wound infection, faster healing, early bowel and bladder recovery, shorter ICU duration, and hospital stay have reduced the patients and families’ economic burden [8]. Based on the established success of ERAS protocols in other surgeries, Hagan et al. in 2016 proposed a set of seventeen evidence-based practices that could be prospectively applied to cater to neurosurgical patients and called upon the necessity of undertaking prospective studies to establish feasibility and success in this field [9]. Since then, there have been very few studies reporting the successful use of ERAS in nonspinal neurosurgery [10].

We describe the use of a modified, multidisciplinary ERAS protocol for neurosurgical patients. This study aims to prospectively analyze the effect of the modified ERAS protocol on the outcomes of patients undergoing elective supratentorial tumor surgeries.

## Methods

### Design and study Centre

Our study was a prospective non-randomized assessor-blinded trial conducted in an 800 bedded tertiary center (training in neurosurgery, neuroanesthesia, and neurocritical care) as the doctoral thesis requirement of A.E. The institute has a dedicated neuro ICU with 24-h coverage by a dedicated team of doctors and nurses. At the time of this study, the area had a combination of 8 ICU and high dependency beds (HDU), and henceforth we use the term ICU to refer to any of these bed types. The study was granted permission by the institute ethics committee (IEC) and was prospectively registered (CTRI/2017/07/015451). Informed written consent was obtained from all patients. A change in the study design (as suggested by the IEC) lead to retrospective re-registration (CTRI/2018/04/013247).

### Patient recruitment

All adult patients of ASA physical status I and II over 18 years (inclusive), with a single supratentorial space-occupying lesion posted for elective craniotomies, were included. Moribund patients requiring emergency craniotomies, uncontrolled diabetics, severely cognitively impaired patients unable to follow simple instructions, and those who did not consent were excluded. Informed consents were obtained, and cohort assignments were based on the patients’ and family members’ agreement to be assigned to the ERAS protocol versus control.

### ERAS protocol vs. conventional care

**Fig. 1:**
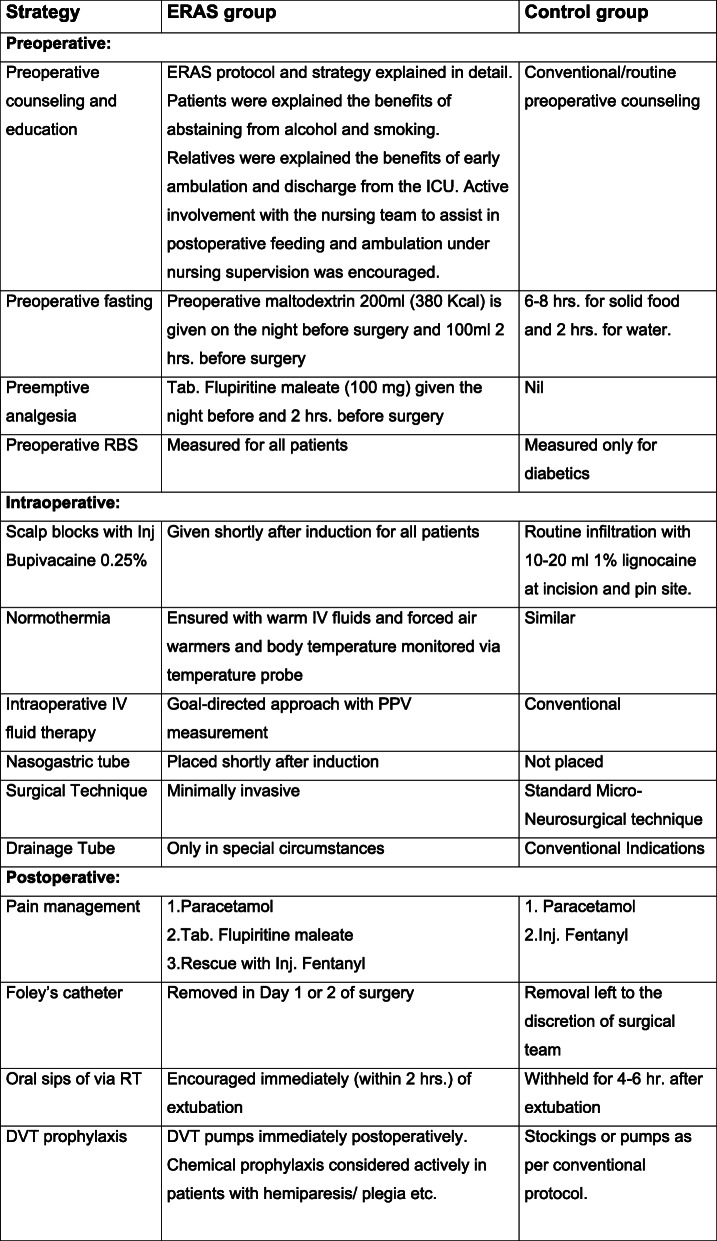
Summary of interventions in the two groups

An ERAS protocol bundle, based on the existing literature and that proposed by Hagen et al., was agreed upon by the Department of Anesthesiology and Critical care and the Department of Neurosurgery in conjunction with the Critical care nursing and dietetics divisions and approved by the institutional ethics committee (vide approval No. IEC/AIIMS BBSR/PG THESIS/2017–18/18). The protocol consisted of primarily three segments – Preoperative, intra-operative, and postoperative. (Fig. 1).

### Conventional care

This group was managed as per existing practices at our center- preoperative fasting, 2 h for clear liquids, and 6 h for solids, and no preemptive analgesics were prescribed. Induction and maintenance of anesthesia were with intravenous propofol (titrated to loss of verbal response), short-acting opioids (fentanyl – 2 mcg/kg body weight), vecuronium (0.1 mg/kg body weight), and isoflurane titrated to minimum alveolar concentration (MAC) of 1.0. Incision site infiltration with lignocaine 2% (10 mL), invasive arterial pressure monitoring with arterial blood gases on demand, point of care sugar monitoring, pulse pressure variation, and temperature management were available to all anesthetists at all times, but their use was not enforced.

Care in the ICU- Standard nurse-led analgesia and sedation management in the ICU followed second hourly documentation of Visual Analogue of pain (VAS) and Richmond Agitation Sedation Scales (RASS), aiming for a VAS < 4 and RASS between − 2 to + 1. Intravenous paracetamol (maximum dose of 1 g per day, unless contraindicated) followed by non-steroidal agents (NSAIDs) as required was administered. Fentanyl (1 mcg/kg body weight) was used only as rescue analgesia for VAS > 4. Dexmedetomidine (first choice) or propofol infusions were used for postoperative sedation of ventilated patients, midazolam being reserved for hemodynamically unstable ones. The unit has standard SOPs for insulin infusion to maintain blood sugars in the 110–180 mg% range. Patients whose blood glucose levels were above 180 mg %were started on an insulin infusion with second-hourly monitoring to reach the target range. Patients meeting pre-set criteria were extubated by the on-floor ICU team of doctors and nurses. However, starting enteral feeds, removing drains, urinary catheters, and ambulation awaited the ICU and neurosurgery consultant rounds the day after the surgery, as per unit protocol.

### ERAS protocol

The preoperative ERAS bundle began in Group ERAS (GrE) with structured preoperative counseling and education. The patients and next of kin were informed about the elements of care of the multimodal ERAS protocol. An active patient and caregiver participation was encouraged to improve compliance. All patients received a preoperative complex carbohydrate maltodextrin drink (Preload®) 100 g in 200 mL of clear water the night before surgery and repeated 50 g in 100 mL water 2 h preceding the surgery [11]. Pre-emptive analgesia was administered- (flupiritine maleate) 100 mg the night before and repeated 2 h before surgery [12]. After induction, all patients received scalp blocks by the anesthetist with 20 mL 0.25% Bupivacaine. Fluid management was based on pulse pressure variation and normothermia ensured by warming blankets. A nasogastric tube was placed in all patients. In ICU, the patients received 100 mg flupiritine maleate eight hourly in addition to the routine protocol. Enteral feeding was started within 6 h of surgery, provided there were no contraindications (such as an anticipated relook surgery). Removal of catheters, drains, and ambulation was directed by a study team member without waiting for consultant rounds: patients who developed urine retention or developed syndrome of inappropriate antidiuretic hormone secretion (SIADH) were re-catheterized on a case to case basis.

### Discharge

The discharge decision from the ICU was taken by the neurosurgical and critical care teams together. Discharge criteria were the same for both groups and included adequate pain control, afebrile state, cardiopulmonary stability, and being able to sit out of bed. Both subsets of patients were followed up till the day of discharge from the hospital.

### Adherence

To maximize adherence, an ERAS checklist was attached to all GrE patient-files after obtaining consent. Before starting the study, a workshop was organized to train the nursing teams of both intensive care units and neurosurgery ward to familiarize the staff and resident doctors with the various elements of the ERAS protocol- this module was repeated twice during the study period to cater to changes in staff- mix.

### Outcome measures and data collected

Duration of ICU stay defined as calendar days from ICU admission was the primary outcome. The secondary measures were total episodes of the visual analogue score (VAS) > 4, insulin (units) and fentanyl (micrograms) administered in the first 48 h of ICU stay, and the total duration of hospital stay after surgery.

All the interventions related to implementing the ERAS protocol were made by the research team belonging to anesthesia (A.E., S.J., S.N.), Critical Care (S.T.), and Neurosurgery (R.N.S). Members of the study team were not involved in the decision to discharge from the ICU or hospital.

All assessments and documentation (pain and sedation scores, analgesic and insulin doses, and stay in ICU and hospital) were made by ICU nurses not a part of the study team. After discharge from the ICU, the ERAS checklist was removed from the patient-files, and a blinded assessor collected the data for analysis. The data analyzer was unaware of the group allocation of the patients.

### Statistics

The sample size calculation was based on data obtained from the previous six months’ medical records. Seventy percent of patients operated for supratentorial tumors by the same surgery, and the anesthesia team had a postoperative ICU stay of > 48 h. It was hypothesized that the ERAS protocol could bring this down by 50%. For 80% power, an alpha error of 5, and 10% attrition, a sample of 70 patients would be needed.

Descriptive statistics were used to compare the patient baseline characteristics. All continuous data which were normally distributed were analyzed using Student’s t-test, whereas nonparametric data were analyzed using the Mann – Whitney U test. A chi-square test or Fisher’s exact test was used for qualitative variables. All the statistical tests were performed using the SPSS software, version 25.

## Results

**Fig. 2:**
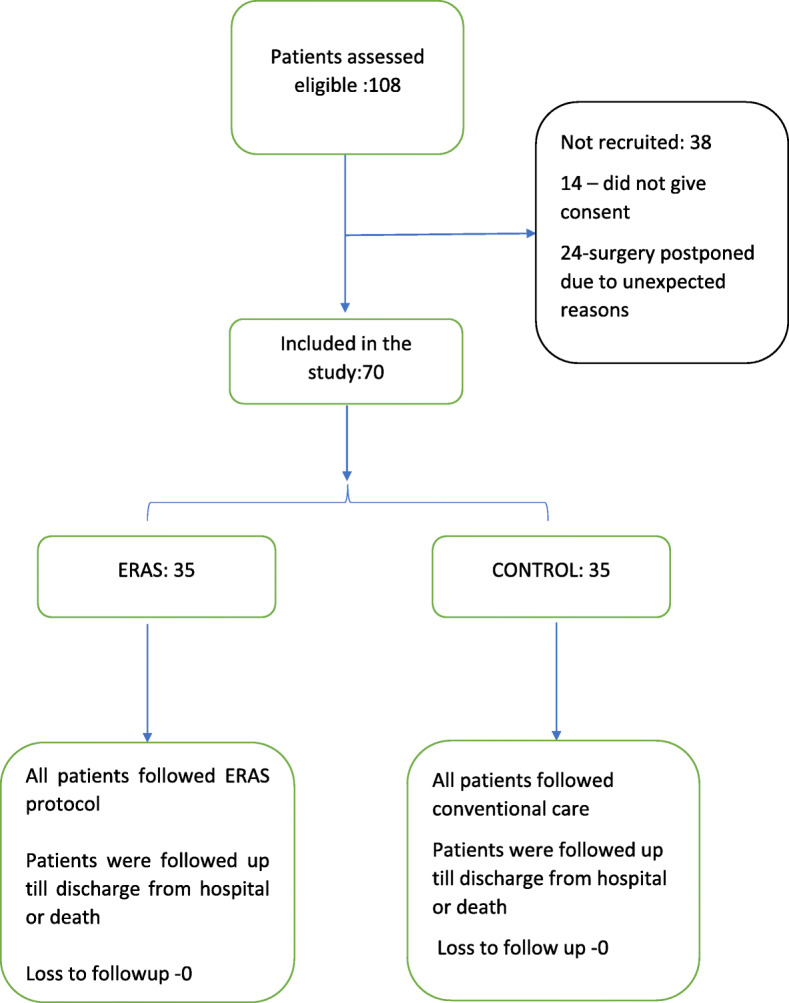
Summary of interventions in the two groups

A total of 108 patients were eligible, of which 14 patients were excluded for lack of consent. Twenty-four patients (11 in Gr E and 13 in Gr C) were excluded after consent due to logistic reasons such as operation theatre unavailability on the day of surgery. Patients were recruited between August 2017 to October 2018, and the follow-up was completed by the end of November 2018. Figure 2.

The baseline demographic characteristics were similar between the two groups (Table 1). The median duration of surgery, intraoperative fluids, urine output, blood loss, and temperature were comparable between groups. The primary indication for surgery did not differ between the groups — the majority of the patients presented with meningiomas, followed by gliomas and craniopharyngiomas. The tumor volume was assessed by the 3-D reconstruction of the C.T. scans during the preoperative period and was similar between the groups. The intra-operative parameters have been summarized in Table 2.

**Table 1:** Baseline characteristics of 70 patient

***Parameter***	***ERAS group (GrE)***	***Control group (GrC)***	***p value***	***CI***
No. of patients (n)	35	35		
Age in years (Median + SD)_	40.89 + 13.61	46.89 + 13.95	0.07	−0.57 – 12.57
Body weight in kg (Mean + SD)_	58.66 + 7.3	60.0 + 6.82	0.43	−2.03 - 4.71
Sex n (%)			0.81	
Males	14 (40%)	16 (45%)		
Females	21 (60%)	19 (55%)		
ASA Classification (n)			1.0	
ASA I	26	26		
ASA II	9	9		
Co- morbid illness (n)				
Hypertension	8	6	0.76	
Diabetes Mellitus	1	3	1.0	

Values have been described in terms of means ± SD for parametric data and medians (range) for non parametric data

**Table 2:** Intraoperative parameters and tumor subtypes in ERAS and the control group

***Parameter***	***ERAS group (GrE)***	***Control group (GrC)***	***p value***	***CI***
No. of patients (n)	35	35		
Nature of tumor (n)			0.72	
Meningioma	19	22		
Glioma	10	9		
Others	6	4		
Duration of surgery (minutes) (Mean + SD)_	253.14 + 72.03	283 + 91.83	0.14	−9.51 - 69.22
Intra-operative blood loss (mL) (Mean + SD)_	824.57 + 518.01	711.43 + 463.06	0.34	− 347.5 - 121.21
Intra-operative crystalloid (mL) (Mean + SD)_	2298.57 + 552.47	2375.71 + 777.28	0.63	− 244.51 - 398.79
Intra-operative temperature (°F) (Mean + SD)_	98.69 + 0.72	98.77 + 0. 82	0.65	−0.28 - 0.45
Tumor volume (mL) Median (range)	45.2 (1090.9)	45.0 (1143.2)	0.70	
Packed RBC volume (mL) (Mean + SD)_	240 + 347.64	190 + 353.51	0.55	− 217.23 - 117.23
Urine output (mL) (Mean + SD)_	685.14 + 393.58	633.14 + 321.31	0.55	− 223.37 - 119.37

(Data have been represented as means ± S.D. or medians (range) and appropriate statistical tests have been used)

Number of patients staying in ICU/HDU for more than 48 h was significantly lesser in the ERAS group of patients than in the Control group. (*X*^2^ (1, 70) = 8.571, *p* = 0.003) The absolute risk reduction was 25.02% (number needed to treat = 4, *CI* = 2.1–52.1), which showed that one out of every four patients benefitted from the ERAS protocol with a reduced ICU stay duration.

Out of 35 patients in Gr E, 21 (60%) had their urinary catheters removed on the first postoperative day against 13 in Gr C. Of the 14 in Gr E whose catheters were not removed, 9 had SIADH.

There was a significant difference in the insulin administered to the groups - Gr C required a higher cumulative dosage of insulin within the first 48 h after surgery (median 0, range 35 Units) than the ERAS group (Median 0, range 12.5 units) (*U* = 486.00, *p* = 0.03, *r* = .25).

Postoperative pain scores showed a significant difference between the groups. Twenty-eight out of 35 patients in ERAS did not have even one episode of VAS above 4 in the first 48 h of ICU stay compared to 19 in the control group. (*X*^2^ (3,70) = 9.79, *p* = 0.02).

The cumulative fentanyl dose in ERAS group (Median 50, range 100 mcg), was significantly lesser as compared to control group (median 50, range 300 mcg) (*U* = 438.50, *p* = 0.02, *r* = .27).

The mean duration of postoperative hospital stay was not different between the two groups (Table 3).

**Table 3:** Primary and secondary outcome measures

***Parameter***	***ERAS group***	***Control group***	***p value***	***CI***
No. of patients (n)	35	35		
Number of patients staying more than 48 h in ICU (n)	15	27	0.003*	
Mean duration of post operative hospital stay (days) (Mean + SD)_	11.49 + 9.04	12.08 + 8.76	0.78	−3.65 - 4.84
Cumulative insulin dose (units) Median (range)	0 (12.5)	0 (35)	0.03*	
Episodes of VAS > 4 in the first 48 h of surgery			0.02*	
0	28	19		
1	7	8		
2	0	7		
3	0	1		
Death / Mortality (n)	2	3	0.6	
Cumulative fentanyl dose (mcg) in first 48 h Median (range)	50 (100)	50 (300)	0.02*	

Data has been represented as means ± SD or median (range) or proportions wherever appropriate

There were three re-explorations (2 decompressive craniectomies for uncontrolled ICP -GrC) and 1 for abscess- GrE); 1 patient developed an infarct (GrC), two patients developed postoperative cerebral edema (GrC), 1 developed transient renal failure (GrE), and 1 went into septic shock (GrE). The ERAS group of patients did not exhibit any complications related to the implementation of the protocol.

## Discussion

There is limited evidence for the benefit of an ERAS protocol in neurosurgery patients. We show in this study that implementing a multidisciplinary ERAS protocol suited for craniotomy patients is feasible and may reduce the proportion of patients requiring a longer ICU stay significantly. We also demonstrate possible associations with specific protocol components responsible for this improvement in the outcome- decreased pain, less requirement of opioids after surgery, better glycemic control, and earlier mobilization.

When our study was initiated in early 2017, there was no evidence that ERAS protocols could be modified and used for neurosurgery patients. A set of suggested guidelines based on non-neurosurgical evidence was presented by Hagan et al., which, although intuitively attractive, would need evidence to be applied to craniotomy patients. ERAS guidelines for spinal surgeries and related studies showing benefits followed [13, 14]. Wang et al. presented the first published evidence for ERAS in craniotomy in 2019, with 140 patients undergoing surgery for both supra and infratentorial tumors [15]. In a series of closely spaced publications (same trial number), this group reported improved benefits in glucose homeostasis, [16] patient satisfaction [17], and decreased postoperative nausea and vomiting (PONV) [18], one retracted since [19]. To the best of our knowledge, at the time of submission, we are the first group from outside of China to report the implementation and benefits of ERAS protocol in craniotomy patients.

ERAS protocols are flexible and adapted to individual centers, keeping the basic tenets in place. This is well demonstrated when the two protocols (ours and Wang et al.) are compared. Many components were based on previous recommendations and were similar between our groups [9].

The differences are as follows-.

Preoperative phase-In our center, relatives are allowed a significant role in patient mobilization, feeding, and patient care - ignorance, fear, and hesitation, delays ambulation, nutritional intake, and rehabilitation during and after the hospital stay. Our preoperative counseling involved an in-depth education of the relatives, including empowering them to question delays in feeding or ambulation orders and removal of foley’s catheter, if any. We educated them on the harmful effects of smoking and alcohol, but abstinence was not essential. As our surgeries were restricted to supratentorial tumors, no added focus than the conventional existed for PONV risk stratification.

We administered maltodextrin (a complex carbohydrate- Proencecarbogain, 100 mg = 380 Kcal) drink in different dosages, volumes and times - 200 ml (100 mg) the night before and 100 ml (50 ml) just 2 h. before surgery vs. Wang’s 400 ml maltodextrose-fructose solution on the morning of the day of surgery.

We had a more aggressive analgesia plan in Gr E in the form of flupiritine perioperatively (no preemptive analgesia by Wang et al.) and bilateral complete scalp blocks along with skin site infiltration (as opposed to only incision site infiltration by Wang et al.). This enabled a stricter postoperative VAS guided pain management plan- paracetamol, tramadol, or NSAIDS up to a VAS of 4 and Inj Fentanyl beyond VAS 4.Unlike Wang et al., we did not emphasize on a difference in the ways of stitching/ craniotomy closure or type of suture used between the two groups. Protocols such as antimicrobial preparation, shaving policies, respiratory interventions, goal-directed therapy, and temperature control were the same between Wang et al. and us.

We found a significant reduction in the proportion of patients requiring an ICU stay of > 48 h, although there was no significant difference in the total duration of hospital stay. The factors affecting the length of postoperative hospital stay are known to vary widely [20]. Ours is a public hospital with highly subsidized charges for patient care: most patients are poor and travel far, with limited post-discharge care. Therefore, our focus was on an early discharge from the ICU/HDU area, which is free and in high demand [21], and our primary outcome pragmatic- reducing the proportion of patients who stayed for > 48 h by 50%.

The ERAS group patients had significantly lower insulin requirements than the controls over 48 h to achieve similar blood glucose targets. Sarin et al. nicely sum the practice points for instituting a protocol for preoperative carbohydrate loading benefits in 2017 [22]. Without having measured serum insulin levels [16], we have demonstrated a reduced stress response and improved postoperative glucose control requiring a lower dose of postoperative insulin.

Scalp blocks provide better attenuation of inflammatory and hemodynamic response to craniotomy and better postoperative analgesia than incision site infiltration with local anesthetics [23]. This, along with the addition of flupiritine, a centrally-acting nonopioid analgesic, with selective action on neuronal potassium channels (Kv7), resulted in significantly improved post-surgical VAS cored in GrE. Flupiritine has been used previously in patients undergoing craniotomy to reduce pain and anxiety perioperatively [12, 24, 25]. As compared to the study by Wang et al., we achieved more stringent goals (VAS < 4) without opioids, and the dose of fentanyl required to manage episodes with VAS > 4 was significantly less in the ERAS group.

The strengths of our study are the inexpensive interventions and the pragmatic reproducible endpoints. ‘Availability’ of family members to care for their patients is common in low, middle-income countries (LMIC) where cultural values and norms encourage family involvement [26]. Many systems utilize this to their strength [27, 28], and we used this concept to ensure adherence to the protocol at all stages, minimizing dropouts.

The lack of randomization is a definite limitation of our study. In 2017, ERAS protocols were allowed to be randomized in gastrointestinal oncosurgeries by our IEC [29]; evidence in craniotomy patients was inadequate to allow randomization, and patients were allowed to chose their treatment arm after full disclosure. Initially, our experience was that getting families and patients to consent to be a part of the study (GrE) was tricky. However, by the first ten patients, word had spread regarding the ‘early’ return of patients to the ward after surgery- then on, getting consent was more straightforward, and we made all attempts to keep the two arms equal by allocating consenting patients sequentially to either group in an attempt to minimize selection bias. As for most clinical research involving surgeries, blinding the patient, surgical or anesthetic teams was impossible.

Notwithstanding clear protocols for pain assessment, analgesia administration, blood sugar control, extubation, and discharge from ICU, members of the surgical, anesthesia, and ICU teams had been educated of the purported ‘benefits’ of ERAS, and bias is possible. All attempts were made to blind the data collector and analyzer, however. The generalizability of our primary results may depend on institute protocols for ICU discharge. We believe that they will be replicable in centers with limited ICU resources (especially if publicly funded) in other parts of the world. The secondary outcomes, based on internationally accepted protocols, should have external validity.

## Conclusion

In this study, we have adapted the ERAS protocol to a neurosurgical setting, showing its feasibility and benefits in early discharge from the ICU/ HDU and better pain and blood sugar control postoperatively. However, there was no difference in length of stay in the hospital. ERAS protocols are bundles of care and include various interventions, and further studies are needed to standardize these for the patients undergoing craniotomy such that they may be kept pragmatic and easy to implement.

## Data Availability

The original data and materials are with the corresponding author and can be obtained on reasonable request.

## Abbreviations

ERAS: Enhanced Recovery After Surgery
ICU: Intensive Care Unit
HDU: High Dependency Unit
IEC: Institute Ethics Committee
CTRI: Clinical Trials Registry of India
ASA: American Society of Anesthesiologists)
SPSS: Statistical Product and Service Solutions
C.T.: Computed Tomography
VAS: Visual Analogue Scale
NSAID: Non-Steroidal Anti-inflammatory Drugs
PONV: Postoperative Nausea and Vomiting
L.A.: Local Anesthetics
LMIC: Low, Middle – Income Countries
